# Filamentous actin disorganization and absence of apical ectoplasmic specialization disassembly during spermiation upon interference with retinoid signaling^†^

**DOI:** 10.1093/biolre/ioaa123

**Published:** 2020-07-17

**Authors:** Sanny S W Chung, Nika Vizcarra, Debra J Wolgemuth

**Affiliations:** 1 Department of Genetics and Development, Columbia University Irving Medical Center, New York, NY, USA; 2 Department of Obstetrics and Gynecology, Columbia University Irving Medical Center, New York, NY, USA; 3 The Institute of Human Nutrition Columbia University Irving Medical Center, New York, NY, USA; 4 The Herbert Irving Comprehensive Cancer Center, Columbia University Irving Medical Center, New York, NY, USA

**Keywords:** filamentous actin disorganization, apical ectoplasmic specialization disassembly, targets for male contraception

## Abstract

Spermiation is a multiple-step process involving profound cellular changes in both spermatids and Sertoli cells. We have observed spermiation defects, including abnormalities in spermatid orientation, translocation and release, in mice deficient in the retinoic acid receptor alpha (RARA) and upon treatment with a pan-RAR antagonist. To elucidate the role of retinoid signaling in regulating spermiation, we first characterized the time course of appearance of spermiogenic defects in response to treatment with the pan-RAR antagonist. The results revealed that defects in spermiation are indeed among the earliest abnormalities in spermatogenesis observed upon inhibition of retinoid signaling. Using fluorescent dye-conjugated phalloidin to label the ectoplasmic specialization (ES), we showed for the first time that these defects involved improper formation of filamentous actin (F-actin) bundles in step 8–9 spermatids and a failure of the actin-surrounded spermatids to move apically to the lumen and to disassemble the ES. The aberrant F-actin organization is associated with diminished nectin-3 expression in both RARA-deficient and pan-RAR antagonist-treated testes. An abnormal localization of both tyrosinated and detyrosinated tubulins was also observed during spermatid translocation in the seminiferous epithelium in drug-treated testes. These results highlight a crucial role of RAR receptor-mediated retinoid signaling in regulating microtubules and actin dynamics in the cytoskeleton rearrangements, required for proper spermiation. This is critical to understand in light of ongoing efforts to inhibit retinoid signaling as a novel approach for male contraception and may reveal spermiation components that could also be considered as new targets for male contraception.

## Teaser

A crucial role of RAR receptor-mediated retinoid signaling in regulating microtubule and actin dynamics in cytoskeleton rearrangements during spermiogenesis is demonstrated.

## Introduction

Mammalian spermatogenesis is a highly organized process with four key transitions: (i) differentiation of spermatogonia, (ii) meiotic initiation, (iii) initiation of spermiogenesis and spermatid elongation, and (iv) release of spermatozoa into the lumen of seminiferous tubules [1, 2]. During spermiogenesis, there is extensive chromatin remodeling and compaction, resulting in the highly condensed nuclei of spermatozoa, which are then released into the tubular lumen during spermiation. Spermiation is a multiple-step process involving profound cellular changes in both spermatids and Sertoli cells, including translocation of spermatids from the adluminal to the luminal compartment, removal of specialized junctions, and eventual disengagement of the spermatids [3].These processes are tightly associated with extensive changes in cellular shape and germ cell movement. The cytoskeleton, which comprises actin, microtubules and intermediate filaments, is believed to function in these cellular events [4–7]. During spermiogenesis, there is extensive restructuring of testis-specific heterotypic adherens junctions, known as the apical ectoplasmic specialization (apical ES) or the Sertoli-spermatid junction, at the interface between spermatids and Sertoli cells [4–6]. This restructuring is essential for the migration of developing spermatids across the epithelium so that fully developed spermatids can be released to the tubule lumen at spermiation.

The apical ES is rich in hexagonal arrays of filamentous actin (F-actin) bundles sandwiched between the Sertoli cell plasma membrane and the endoplasmic reticulum. During spermiation, apical ES disassembly occurs—a gradual process that begins during late stage VII and involves reorganization of F-actin bundles into a highly branched network, which in turn facilitates spermatid movement and proper orientation [3]. Microtubules are also important components of the Sertoli cell cytoskeleton [4, 7]. A microtubule-directed mechanism has been suggested to also be involved in translocation of mature spermatids to the lumen [8]. Intratesticular injection of taxol, a microtubule stabilizer, led to the retention of rat step 19 spermatids deep within Sertoli cells crypts within 12–24 h, instead of their being transported toward the tubular lumen [8]. This suggested that microtubules also play an integral role in the spermatid translocation toward the tubular lumen.

It has been known for almost a century that signaling through vitamin A metabolites is indispensable for spermatogenesis [9–13]. Disruption of retinoic acid receptor alpha (RARA) function [13–17] and treatment with a pan-RAR antagonist [18, 19] resulted in male sterility and aberrant spermatogenesis, which resembled vitamin A deficiency (VAD) [20]. In particular, we have observed that spermiation in stage VII and VIII tubules is one of the physiological processes in spermatogenesis that is defective in all conditions of disrupted retinoid signaling including VAD, treatment with a pan-RAR antagonist, and disruption of RARA function. They include defects in the orientation of step 8–9 spermatids with regard to the basal aspect of Sertoli cells, a failure of spermatid alignment at the lumen in stage VIII tubules, and defects in spermiation [13, 15–20]. Interestingly, RARA mRNA and protein levels have been shown to be the highest at stage VIII in both germ cells and Sertoli cells [21, 22] and the levels of all-*trans*-retinoic acid (ATRA) are highest in these stages as well, as measured by HPLC analysis of synchronized tubules [23]. A recent study has further shown that ATRA plays primary roles at two postmeiotic transitions: the initiation of spermatid elongation and spermiation [24]. After injection of the inhibitor WIN18,446, both spermatid elongation and spermiation were delayed, and conversely, a single injection of RA was sufficient to precociously induce both these transitions [24, 25]. However, neither the cellular nor molecular basis for this phenotype has been elucidated. Moreover, it is unknown what cellular structures involved in spermiation in the tubules are affected in the absence of RAR receptor-mediated retinoid signaling.

In the present study, we provide evidence for distinct functions of RAR-mediated retinoid signaling in regulating the changes in the cytoskeleton required for proper spermiation. We demonstrate that defects in spermiation are among the earliest abnormalities in spermatogenesis upon pharmacologic inhibition of retinoid signaling. Further, F-actin disorganization and an absence of apical ES disassembly were observed as well as disruption of the proper cellular localization of tyrosinated and detyrosinated tubulins during spermatid translocation. These results revealed a critical role for RAR receptor-mediated retinoid signaling in regulating microtubule and actin dynamics during the cytoskeleton rearrangements, which in turn control proper spermiation, and hence, male fertility. Understanding the basic biology of cellular structures involved in defects in spermiation and their related cytoskeletal elements in Sertoli cells could also lead to the identification of new molecular targets for the development of male contraceptives.

## Materials and methods

### Source of animals and tissue processing

The use of animals was approved by Columbia University Medical Center’s Animal Care and Use Committee. The generation of *Rara−/−* mice has been described previously [14]. CD1 mice (8 weeks, 30 g body weight) were obtained from Charles River Laboratories. Testes were dissected from anesthetized drug-treated 8-week-old CD1 mice at the specified time-points or from young adult (8 weeks) *Rara−/−* mice and weighed. One testis was fixed with 4% paraformaldehyde in 1x phosphate buffered saline (PBS) buffer and the second with Bouin’s fixative overnight at 4°C. Fixed tissues were embedded in paraffin or frozen in liquid nitrogen, sectioned at 5 μm, and mounted on Superfrost slides (Fisher) as previously described [17, 18, 26]. For staging of testicular tubules [2], sections were stained for the Periodic acid-Schiff reaction before hematoxylin counterstaining as previously reported [15, 16] and examined by bright-field microscopy. Rhodamine-conjugated peanut agglutinin (PNA) diluted in PBS was used to detect acrosomes in fluorescent staining (Vector Laboratories, RL-1072) as described previously [27, 28]. The staging of tubules was analyzed according to the criteria developed by Oakberg [1] and refined by Russell et al. [2]. The abnormal approximately staged tubules in *Rara−/−* and pan-RAR antagonist-treated seminiferous tubules have been designated with a Roman numeral followed by an asterisk (e.g., stage IX^*^) as previously reported [15–19].

### Treatment of mice with a pan-RAR Antagonist, Compound 9

Oral delivery of the drug to CD1 mice followed our previously described protocols [18, 26]. Briefly, the pan-RAR antagonist, Compound 9 (the commercially synthesized version of BMS-189453) [18], was suspended in a vehicle of aqueous 1.5% Avicel (CL-611, FMC BioPolymer) to obtain the desired concentrations and was administered to CD1 males (*n* = 5 each group) via oral gavage with a standard daily dose of 5 mg/kg/day for time periods as specified. Control groups (*n* = 5) received vehicle alone for 7 days. During the period of treatment, the mice were observed daily for changes in overall health and behavior as described previously [18, 19].

### Time course study

To assess the effects on spermiation during the course of the standard 7 day treatment regimen with the pan-RAR antagonist, 8-week-old mice (*n* = 5/time point) were treated with vehicle alone or at 5 mg/kg/day in vehicle by oral gavage and terminated 1 day after dosing periods of 1 to 7 days. To quantify the abnormalities within testicular tubules at the histological level, five males were used and 100–150 tubules per testis were analyzed. As described previously, only round-shaped tubules were assessed [15, 16]. Statistically significant differences were assessed using Student’s paired *t*-test using GraphPad Analysis software.

### Immunohistochemistry, immunofluorescence, and F-actin staining

Paraffin and frozen sections of mouse testes were immunostained using a Vectastain ABC kit (Vector Laboratories, Burlingame, CA) as previously described [17, 29]. The following primary antibodies were used: rat monoclonal Anti-nectin 3 (1:50; #103-A1, Hycult Biotech, Netherlands); mouse monoclonal anti-tyrosinated tubulin (1:1000, TUB-1A2, Sigma Aldrich) and rabbit polyclonal anti-detyrosinated tubulin (also referred to as glu-tubulin, reflecting the newly exposed C-terminal glutamate residue) [1:400; gift from the Gundersen Lab at Columbia University] [30, 31]. For controls, the slides were incubated with the appropriate immunoglobulin G (IgG) or pre-immune serum instead of primary antibody. For comparison of the relative levels of expression between samples, caution was made to minimize the variation in fixation, thickness of sections, etc. by for example, samples being processed at the same time and obtaining standard 5 μm-thicknesses for the sections. In addition, sectioned tissues from different experimental groups (for example, control versus drug-treated, etc.) were collected onto a single slide to enable relative quantitative comparisons with greater confidence [16, 32]. The sections were viewed on a Nikon photomicroscope under bright-field optics.

For immunofluorescence, slides were blocked with universal blocking solution (CAS-Block, Invitrogen) for 30 min. The secondary antibodies used were diluted to 1:200 in CAS-Block: Alexa 594-conjugated goat anti-rat IgG (H + L) (Invitrogen, A-11007). For F-actin staining, slides with frozen sections of testes were first incubated with Image-iT FX Signal Enhancer (Invitrogen Molecular Probes) for 30 min and then Alexa Fluor 488 Phalloidin (A-12379, Invitrogen Molecular Probes) diluted in 1x PBS with 1% BSA for 20 min. Slides were counterstained with DAPI, mounted with Glycergel mounting medium, and viewed on a Nikon Eclipse 800 photomicroscope under fluorescent-field optics. Each experiment had *n* = 3 independent samples, which yielded similar results, and was performed at least twice. For the specific staining and distribution of cells in the seminiferous tubules, at least 100 histological sections of tubules were randomly selected and examined in both experimental and control groups. Data shown herein were representative photomicrographs from a single experiment. Fluorescence intensity was analyzed using the public domain software ImageJ (NIH).

### Confocal microscopy and co-localization studies

To confirm the localization of tyrosinated tubulin and detyrosinated tubulin, slides of 25 μm-sections were blocked with 5% donkey serum for an hour, followed by the dual-labeled immunofluorescent staining. The following primary antibodies were used: mouse monoclonal anti-tyrosinated tubulin (1:1000) and rabbit polyclonal anti-detyrosinated tubulin (1:400). After rinsing in PBST (phosphate-buffered saline, 1% Tween-20), the slides were incubated with the appropriate secondary antibodies diluted 1:200 in PBS as follows: Alexa 488-conjugated goat anti-mouse IgG (H + L) (Invitrogen, A-11029), and Alexa 647-conjugated donkey anti-rabbit IgG (H + L) (Jackson ImmunoResearch, 711–605-152). Rhodamine-conjugated PNA at 1:2000 dilution in PBS was used to detect acrosomes (Vector Laboratories, RL-1072) as described previously [19, 27, 28], counterstained with DAPI, and mounted with Glycergel mounting medium. Slides were first viewed on a Nikon photomicroscope under fluorescence optics. Fluorescent images were captured using a confocal Zeiss LSM710 microscope as described previously [33, 34]. Z stacks, composed of 0.25-μm optical sections, were obtained using the Zen 2011 software (Zeiss). Images were subsequently analyzed and processed using the public domain software ImageJ (NIH).

## Results

### Defects in spermiation upon inhibition of retinoid signaling

**Figure 1:**
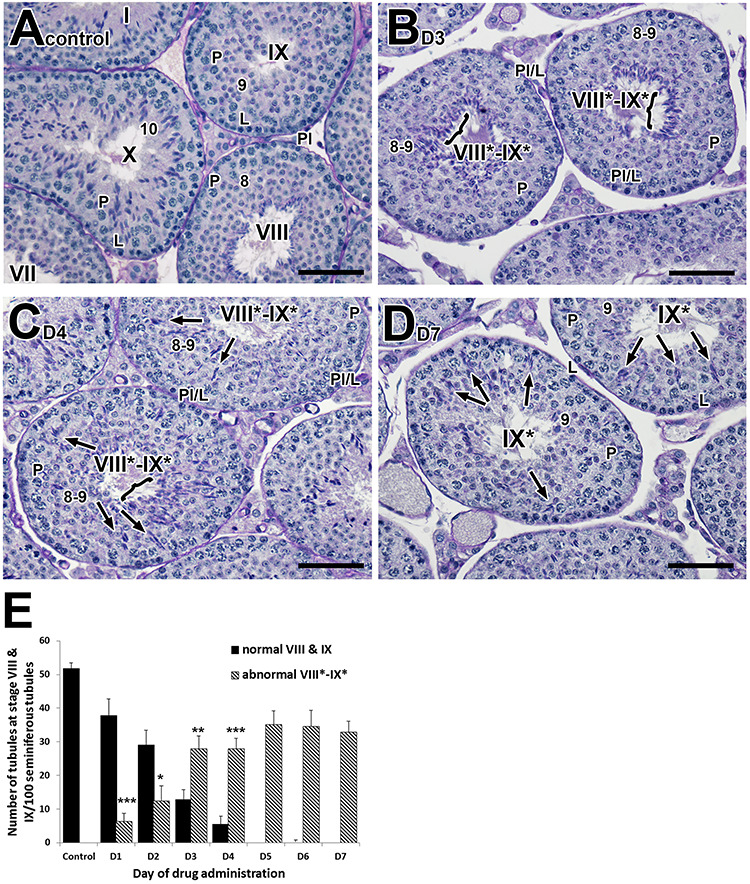
Failure of spermatid translocation and sperm release commences very early after drug treatment. (A−D): Histological sections of testes from 8-week-old adult mice treated with vehicle alone and 5 mg/kg of pan-RAR antagonist in vehicle for 3, 4, 7 days, respectively, and examined one-day after cessation of treatment. (A−D): Original magnification, ×40. (E): Bar graph showing the number of both normal and abnormal stage VIII and IX tubules in control and pan-RAR antagonist-treated males. The total number of stage VIII and IX tubules per 100 seminiferous tubules was quantified (five mice for each dosing regimen) at different dosing periods (D1 to D7 of drug administration). The error bars represent the mean ± SD of the counts. ^*^^*^^*^, P < 0.001; ^*^^*^, P < 0.01 and ^*^, P < 0.05. Roman numerals indicate the stage of the tubules. The abnormal approximately staged tubules are labeled with a Roman numeral followed by an asterisk (e.g., stage IX^*^). L, leptotene spermatocytes; P, pachytene spermatocytes; Pl, pre-leptotene spermatocytes; Pl/L, pre-leptotene/leptotene spermatocytes. Scale bar, 50 μm.

We had previously reported the observation of spermiation defects, including defects in spermatid orientation, translocation and release, in both RARA-deficient [14–16] and pan-RAR antagonist-treated models [18, 19]. To detect how soon we observed the defects after administration of the pan-RAR antagonist, we examined systematically the effects on spermiation during the course of the standard 7-day treatment regimen. Specifically, 8-week-old mice (*n* = 5/time point) were treated at 5 mg/kg/day and euthanized at different time points during the dosing period. To quantify the abnormalities within testicular tubules at the histological level, three to five males were used and 100–150 tubules per testis were analyzed. Interestingly, as compared to control (Figure 1A), we found that defects in spermiation (Figure 1B−D) are indeed among the earliest abnormalities in spermatogenesis observed in response to treatment with the pan-antagonist (Figure 1E). To address which aspects of spermiation, whether a failure of spermatid translocation or a failure of spermatid release, were first induced in response to treatment, we examined carefully the testes after 3, 4, and 7 days of drug treatment. Strikingly, a failure of step 16 spermatid release or spermatid disengagement (the final stage of spermiation) was observed as early as 3 days of treatment with the pan-antagonist (Figure 1B). After 4 days of pan-antagonist treatment, both failure of spermatid translocation and release were observed in drug-treated testes (Figure 1C), and after 7 days of pan-antagonist treatment, both failure of spermatid translocation and release were consistently observed (Figure 1D). This suggests that at day 3 post treatment, although the early proper alignment of step 16 elongated spermatids at the tubular lumen of stage VIII tubules was seen, they fail to disengage at spermiation.

### Filamentous actin (F-actin) disorganization and failure of apical ES disassembly

**Figure 2:**
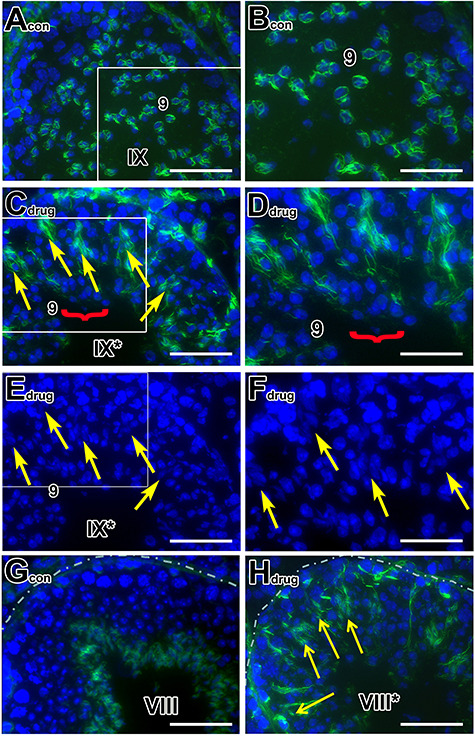
Aberrant location of F-actin surrounding the retained late elongated spermatids at stage IX and VIII tubules of pan-RAR antagonist-treated testes. (A–H): Histological sections of testes from 8-week-old adult control (A, B, G) and pan-antagonist-treated (C-F, H) mice with 5 mg/kg for 7 days and examined one-day after treatment. The F-actin bundles of the ES are labeled with fluorescent dye-conjugated phalloidin (green) and counterstained with nuclear DAPI (blue). (A−H): Original magnification, ×60. (A−D, G and H**)**: merged image of F-actin and DAPI and (E and F): DAPI staining showed the same tubule in (C), respectively, for displaying retained spermatids. Arabic numerals indicate the step of spermatid differentiation. Roman numerals indicate the stage of the tubules. Abnormal tubules are labeled with a Roman numeral followed by an asterisk (e.g., stage IX^*^). The yellow arrows in (C, E, and F**)** point to the abnormally retained elongated spermatids while the red bracket in (C) and (D) indicates step 9 spermatids. (B, D, F) are the magnified inset of (A, C, E), respectively. Scale bar in (A, C, E, G, and H), 50 μm; scale bar in inset (B, D, F), 80 μm.

Given the important role of F-actin in proper spermiogenesis [3–7], we wished to determine whether abnormalities in formation of F-actin bundles might be involved in both pan-RAR-treated and *Rara−/−* testes. We therefore used staining with AF488-conjugated phalloidin (green) to visualize the structures in control versus the experimental testes. In control mouse testes, we observed a stage-specific, spatial localization of F-actin bundles at the apical ES between elongating spermatids and Sertoli cells in the seminiferous tubules (Supplementary Figure S1A−H and Figure 2A, B, and G), consistent with observations reported by others [35]. Specifically, F-actin bundles were first detected in step-9 spermatid heads at stage IX in a crescent overlying the convex aspect of the acrosomal region (steps 9–12) (Supplementary Figure S1G; and at higher magnification, Figure 2A and B) and later surround the closely clustered step 13–15 spermatids deep in crypts of the Sertoli cells at stages I−V (Supplementary Figure S1A−C). During spermiation, F-actin bundles are located along the luminal surface in late elongated spermatids (step 16; stages VII and VIII), which have been translocated to the adluminal compartment (Supplementary Figure S1D and E; Figure 2G). F-actin was no longer detectable at Sertoli-spermatid junctions at the end of stage VIII, after apical ES disassembly to permit disengagement of spermatids (Supplementary Figure S1F).

In both pan-antagonist-treated (Figure 2C−F, H) and *Rara−/−* (Supplementary Figure S2C−F) testes, F-actin was abnormally distributed and bundles failed to form at the apical ES. For example, in contrast to the localization of F-actin in a crescent overlying the convex aspect of the acrosomal region as shown in control testes (Figure 2A and B), there was no localization of F-actin at the apical ES of step 9 spermatids (Figure 2C and D, red bracket), indicating the apical ES was not properly established at the interface between the elongating spermatids and Sertoli cells. Interestingly, F-actin was found surrounding the abnormally retained condensing elongated spermatids at stage IX, suggesting that apical ES disassembly does not occur (Figure 2C−F, yellow arrows). This suggested a distinct effect of the pan-RAR antagonist on overall F-actin dynamics; that is, it appears to block formation of new ES junctions (red brackets) and block disassembly of ES junctions on elongated spermatids (yellow arrows). In addition, we observed F-actin surrounding aberrantly retained spermatids at stage VIII^*^ (Figure 2H, yellow arrows), as compared to the F-actin bundles located along the luminal surface in late condensing stage 16 spermatids that had translocated from the adluminal compartment in control testes (Figure 2G).

### F-actin disorganization is associated with diminished nectin-3 expression in *Rara−/−* testes and pan-antagonist-treated testes

It has been shown that two major adhesion molecules in the testes, nectin-2 in Sertoli cells and nectin-3 in spermatids, together with the adaptor protein afadin, co-localize with F-actin at apical ES junctions [35]. In nectin-2 null testes, spermatids were randomly distributed within the tubules, with abnormally positioned afadin and F-actin bundles, suggesting defects in the apical ES [35]. Further, F-actin was disorganized and nectin-2 was absent in nectin-3 null testes indicating that the presence of nectin-3 is essential for the localization of nectin-2 at apical ES as well [36].

**Figure 3:**
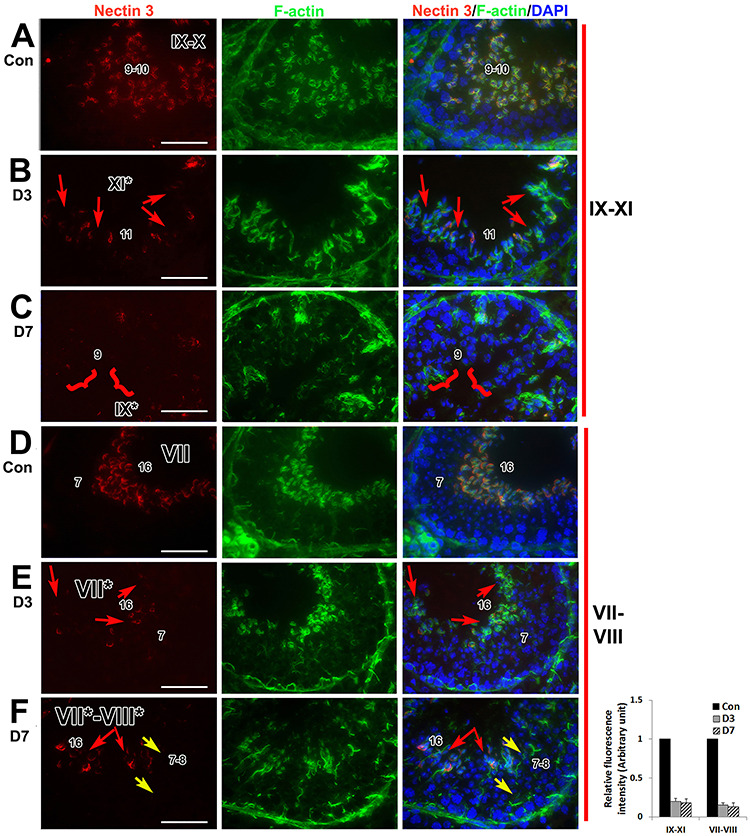
The F-actin disorganization associated with downregulation of nectin-3 in pan-RAR antagonist-treated testes. (A, D): Histological sections of testes from 8-week-old adult wild-type control mice treated for 7-days with vehicle alone. (B and C, E and F): Histological sections of testes from 8-week-old mice treated with pan-RAR antagonist at 5 mg/kg for 3 days or 7 days and euthanized one-day after dosing. (A−F): Original magnification, ×60. Left panel: localization of nectin-3 (red); middle panel: localization of F-actin (green); right panel: co-localization of nectin-3 (red), F-actin (green) and DNA (blue). The Arabic numerals indicate the step of spermatid differentiation. The red arrows in (B, C, E) point to the spermatids without nectin staining while the yellow arrows in C point to the retained spermatids without nectin-3 staining. The red brackets in (F) point to the step-9 spermatids without nectin-3 staining. A graph showing the relative fluorescence intensity of nectin-3 staining in drug-treated vs. the control vehicle group, illustrating an ~ 80% downregulation. Scale bar, 50 μm.

We first confirmed the reported stage-specific co-localization of nectin-3 and F-actin at the apical ES in control testes [35] (Supplementary Figure S3A and B). F-actin (green) and nectin-3 (red) co-localize in step-9 spermatid heads at stage IX (Supplementary Figure S3B). During spermiation, at stage VII, the step 16 condensing spermatids are translocated to the luminal surface of the seminiferous tubules. Nectin co-localized with F-actin at apical ES of the step 16 spermatids, which were aligned at the luminal surface of the stage VII tubules (Supplementary Figure S3A). In *Rara−/−* testes (Supplementary Figure S3C and D), however, the detection of nectin-3 was reduced or absent in the elongating spermatids (step 9–11 spermatids) at stages IX^*^−XI^*^ (Supplementary Figure S3D, red brackets), as compared to control (Supplementary Figure S3B). The lack of nectin-3 expression became more prominent during spermiation. At stage VII, absence of nectin-3 expression was found in retained elongated spermatids (yellow arrows in Supplementary Figure S3C), which were located adluminally rather than aligning at the tubular lumen. Interestingly, we detected downregulation of nectin-3 in concomitant preliminary microarray analysis of *Rara−/−* testes (data not shown),

We next examined whether levels of nectin-3 were also downregulated in the pan-RAR antagonist-treated testes as assessed by immunohistochemistry. Indeed, as compared to control (Figure 3A), the detection of nectin-3 was reduced in elongating spermatids (step 9–11, stages IX^*^−XI^*^) after 3 days of treatment (Figure 3B, red arrows) and by 7 days after treatment, most elongating spermatids lacked detectable nectin-3 expression (Figure 3C, red brackets). Downregulation of nectin-3 expression was consistently found during early stages of spermiation (Stages VII^*^ and VIII^*^, Figure 3E and F) as compared to control (Figure 3D). Three days after drug treatment, an absence of nectin-3 expression was found in most of the condensing elongated spermatids in stage VII^*^ tubules, although these elongated spermatids were properly translocated to the adluminal compartment and were aligned at the luminal surface of the tubules (red arrows in Figure 3E). By 7 days after treatment, at stage VII^*^ to VIII^*^, nectin-3 expression was not detected in either retained (Figure 3F, yellow arrow) or translocated (Figure 3F, red arrow) elongated spermatids in stage VII^*^ and VIII^*^ tubules. Therefore, pan-RAR antagonist-induced spermatid retention is associated with diminished nectin-3 at the Sertoli-spermatid junctions and with F-actin disorganization.

### Abundant tyrosinated tubulin surrounding the aberrantly retained spermatids at stages VII^*^−IX^*^ in *Rara−/−* testes and pan-RAR antagonist-treated testes

Mature spermatids are thought to be translocated to the lumen by Sertoli cells via the movement of junction plaques along a microtubule tract by minus-end-directed dynein motor proteins [37–40]. We hypothesized that retinoid signaling could affect microtubule dynamics, specifically involving alteration of the levels of tyrosinated tubulin, which is highly expressed during tubulin polymerization, and detyrosinated tubulin, which is found in stabilized microtubules and shown to be important for anchoring elongating spermatids into the deep crypts of Sertoli cells at stages IX−XII [31, 41, 42].

**Figure 4:**
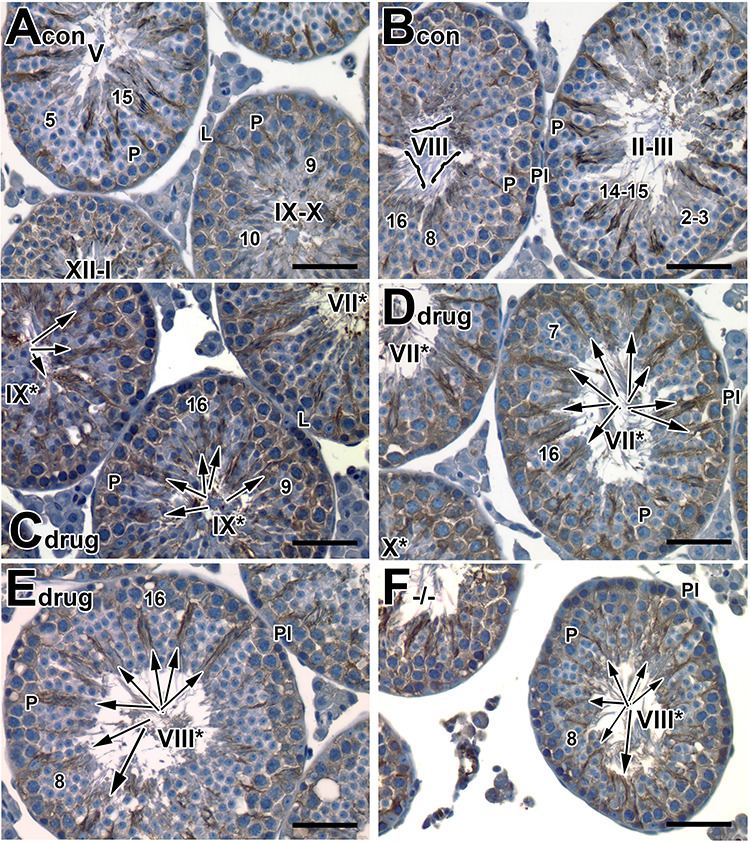
Tyrosinated tubulin surrounding the aberrantly retained spermatids at stages VII^*^−IX^*^ in *Rara−/−* testes and pan-RAR antagonist-treated testes. (A–F): Histological sections of testes from 8-week-old adult control (A, B), pan-antagonist-treated (C, D) mice with 5 mg/kg for 7 days and examined one-day after treatment, and *Rara−/−* (E, F) testes. Sections were stained with an anti-tyrosinated tubulin antibody to detect tubulin polymerization. Original magnification, ×40. Pl, Preleptotene spermatocytes; L, leptotene spermatocytes; P, pachytene spermatocytes. The Arabic numerals indicate the step of spermatid differentiation. Roman numerals indicate the stage of the seminiferous tubules. Although abnormal cell associations complicate staging, an attempt was made to stage the pan-antagonist-treated tubules using acrosomal system, and tubules are labeled with a Roman numeral followed by an asterisk (e.g., stage IX^*^). The arrows in (C−F) point to the retained spermatids with tyrosinated staining. The brackets show that the staining on normal spermatids at stage VIII was weakly and sparsely detected. Scale bar, 50 μm.

We first asked if there was a stage-specific and restricted spatial localization of tyrosinated tubulin in mouse testes, similar to that described in rats [43] (Figure 4A and B). At stage IX-stage I, branched localization of tyrosinated tubulin was observed surrounding pachytene spermatocytes (Figure 4A; Supplementary Figure S4A). Tyrosinated tubulin also surrounded elongating spermatid heads (step 9–12) in a crescent overlying the convex aspect of the acrosomal region, which allows the establishment of microtubules (tubulin polymerization). Once condensing spermatids (step 14–15; stages II−VI) were in the crypts of Sertoli cells, tyrosinated tubulin became condensed into a spoke-like pattern (Figure 4A and B; Supplementary Figure S4B), indicating active tubulin polymerization, which is critical for spermatid translocation and spermiation. During stages VII and VIII, when spermatids are translocated to the luminal surface, the spoke-like pattern of tyrosinated tubulin was weakly and sparsely detected (Figure 4B, bracket; Supplementary Figure S5A). This reduced tubulin polymerization (reflected by reduced tyrosinated tubulin) was observed at stages VII and VIII, when the spermatids have already been translocated to luminal surface and the microtubule tracts are no longer needed.

In testes of mice treated with the pan-RAR antagonist, tyrosinated tubulin was ectopically localized around the aberrantly retained elongated spermatids at stage VIII^*^ and IX^*^ tubules (Figure 4C−E, arrow; Supplementary Figure S5B), which was never seen in control step 16 spermatids (Figure 4B, bracket). A similar pattern of ectopic localization of tyrosinated tubulin was seen in the *Rara−/−* testes (Figure 4F, arrow), with tyrosinated tubulin surrounding the retained late condensing elongated spermatids at stages VIII^*^ and IX^*^. This suggested that elongated spermatids were retained in the adluminal compartment and were not translocated along the microtubule tracks to the luminal surface for spermiation, leading to persistence of tyrosinated tubulin at stage VIII^*^ tubules in *Rara−/−* testes (Figure 4F), as compared to control (Figure 4B, stage VIII tubule).

**Figure 5:**
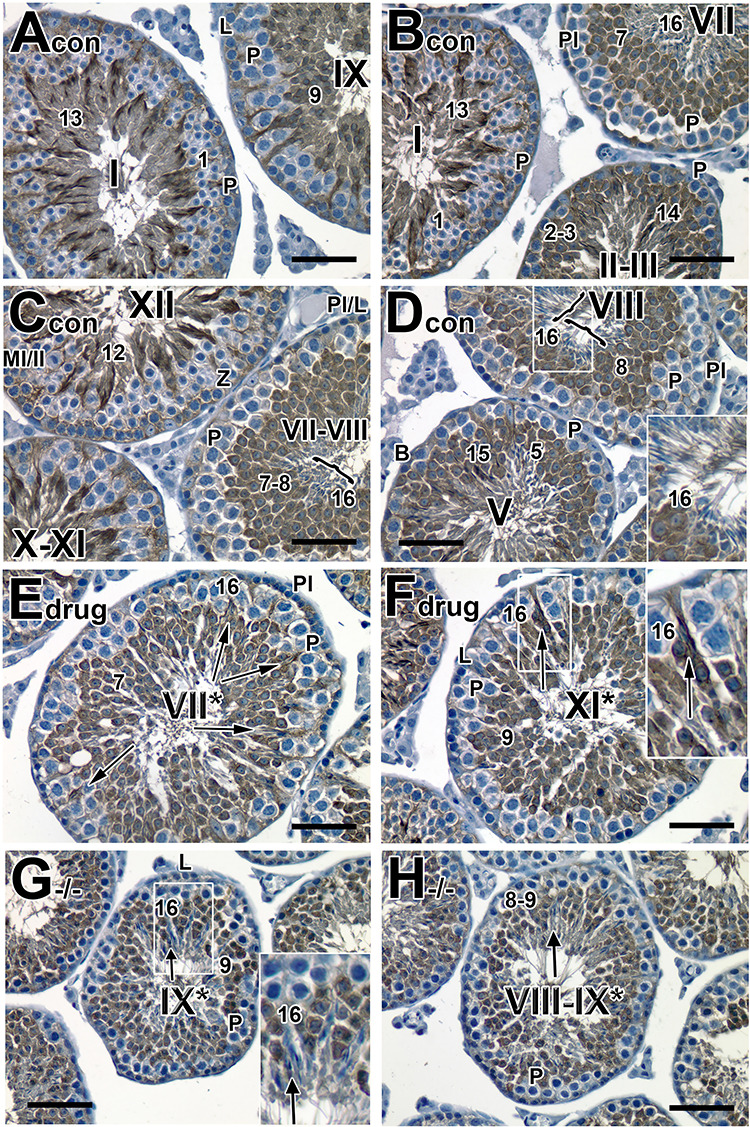
Aberrantly retained spermatids surrounded with detyrosinated tubulin at stage VII^*^–IX^*^ tubules in pan-RAR antagonist-treated testes. (A–H): Histological sections of testes from 8-week-old adult control (A–D), pan-RAR antagonist-treated (E, F) mice with 5 mg/kg for 7 days and examined one-day after treatment, and *Rara−/−* (G, H) testes. Sections were stained with an anti-detyrosinated tubulin antibody to label tubulin polymerization. Original magnification, ×40. The arrows in (E) and (F) point to retained spermatids with detyrosinated tubulin, but not in (G) and (H). The brackets in (C) and (D) show that the staining on normal spermatids at stage VIII was very weak. Pl, Preleptotene spermatocytes; L, leptotene spermatocytes; Z, zygotene spermatocytes; P, pachytene spermatocytes. The Arabic numerals indicate the step of spermatid differentiation. The Roman numerals indicate the stage of the seminiferous tubules. Although abnormal cell associations complicate staging, an attempt was made to stage the pan-RAR antagonist-treated tubules using acrosomal system, and tubules are labeled with a Roman numeral followed by an asterisk (e.g., stage IX^*^). The inserts in (D, F) and (G) displayed the enlarged magnification of the corresponding figures. Scale bar, 50 μm.

**Figure 6:**
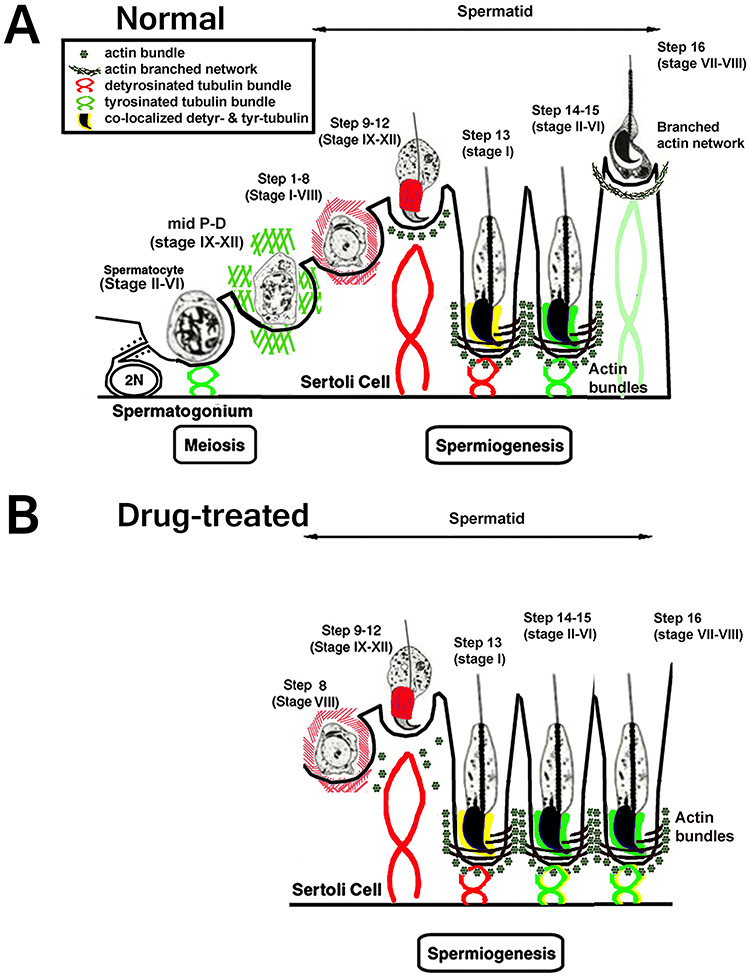
A cartoon illustrating the changes in cellular localization of tyrosinated (green) and detyrosinated (red) tubulins and actin dynamics during spermatid translocation in the seminiferous epithelium of normal (A) and drug-treated testes (B).

### Abundant detyrosinated tubulin surrounds retained spermatids in testes with disrupted retinoid signaling

In control testes, detyrosinated tubulin (reflecting stabilized microtubules) was detected from stage IX to XII, surrounding elongating spermatid heads (step 9–12) predominantly within the manchette region (Figure 5A−C, Figure 6A, red), which was confirmed with confocal microscopy (Supplementary Figure S4A). A condensed, spoke-like pattern of detyrosinated tubulin was found as a bundle in Sertoli cells surrounding leptotene, zygotene, and diplotene spermatocytes from stage IX to XII and pachytene spermatocytes from stage IX-I (Figure 5A−C; Figure 6A, red). At stage I, there was robust detyrosinated tubulin expression surrounding step 13 condensing spermatids (Figure 5A and B, Figure 6A, red), indicating that tubulin polymerization was stabilized so as to anchor condensing spermatids into the crypts of Sertoli cells. Interestingly, once the spermatids (step 14–15; stages II−VI) are clustered together, detyrosinated tubulin (normally detected in stabilized microtubules) was no longer detected in a spoke-like pattern around step 14–15 spermatids nor around step 16 spermatids and was very weak (bracket, stage VIII, Figure 5C and D; Figure 6A, red; Supplementary Figure S5A). It was, however, detected surrounding step 2–8 round spermatids during stages II−VIII (Figure 5B−D; Figure 6A, red). Interestingly, this localization is in contrast to tyrosinated tubulins: once the condensing spermatids (step 14–15; stages II−VI) are clustered together, tyrosinated tubulin was strongly expressed (Figure 4A and B, Figure 6A, green; Supplementary Figure S4B). Before and during spermiation, the spoke-like pattern of detyrosinated tubulin was barely detectable around step 16 spermatids at stages VII and VIII (Figure 5C and D, inset in Figure 5D).

In pan-RAR antagonist-treated testes, detyrosinated tubulin was ectopically localized around the aberrantly retained late elongated spermatids at the basal region in stages VII^*^–IX^*^ tubules (Figure 5E and F, arrow; Figure 6B, red). This observation was also confirmed with confocal microscopy (Supplementary Figure S5B) and is quite different from the complete lack of detyrosinated tubulin distribution surrounding step 16 spermatids in the control testes (Figure 5D). Since detyrosinated tubulin is found in stabilized microtubules, we suggest that these retained elongated spermatids failed to translocate during stages VII^*^ and VIII^*^ because of its persistent presence. Interestingly, the retained spermatids with abundant detyrosinated tubulin were only observed in the pan-antagonist-treated testes, but not in the retained spermatids in *Rara−/−* stage IX^*^ tubules (Figure 5G and H, arrow).

## Discussion

We have previously shown that *Rara−/−* testes and pan-RAR antagonist-treated testes exhibit failure of spermatid alignment and sperm release. In the present study, we extended this observation to a more mechanistic level, illustrating distinct functions of RAR-mediated retinoid signaling in regulating microtubule and actin dynamics during the cytoskeleton rearrangements that are required for proper spermiation. We first characterized the time course of appearance of spermiogenic defects in response to treatment with the pan-RAR antagonist. The results revealed that defects in spermiation, especially in release of spermatids, are indeed one of the earliest abnormalities in spermatogenesis observed upon inhibition of retinoid signaling. This is similar to spermiation failure induced by gonadotrophin suppression model [44], in which spermiation failure occurs as a result of a defect in the final release or disengagement of spermatids, and not defects in earlier spermiation processes [44]. The proteins that maintain the spermatid and the Sertoli cell junctions until the point of disengagement have been suggested to be one of the critical candidates in regulating sperm release [3]. These proteins include adhesion-related proteins such as α6β1 integrin and galectin 1, signaling proteins (including phosphorylated FAK, SRC, CDC42, CSK, PTEN, PI3kinase, and ERK) and proteases, such as MT-MMP1 and MMP2 [3]. Interestingly, in studies on adjudin functions, sperm release at spermiation was reported to be regulated by changes in the organization of actin and microtubule-based cytoskeletons at the apical ES [45] and regulation of spermatid polarity by the actin- and microtubule-based cytoskeletons was also suggested [46]. Understanding the basic biology of cytoskeletal structures involved in defects in spermiation not only provides insight into the underlying causes of male infertility but could also lead to the identification of new molecular targets for development of male contraceptives.

Among the more striking sub-cellular aspects of the spermiation defects exhibited by the knockout and pan-RAR antagonist models were the F-actin disorganization and an absence of apical ES disassembly (summarized in the cartoon in Figure 6). Apical ES disassembly is indeed a crucial step during spermiation. In particular, in early stage VIII, actin-binding proteins (e.g., EPS8 and palladin) are downregulated while actin-branching proteins (the ARP2/3 complex) are induced [3–6]. As such, F-actin changes from a bundled to branched configuration and destabilizes the adhesion protein complexes. In addition, it has been reported that the nonreceptor protein tyrosine kinases (e.g., c-YES and c-SRC) also facilitate endocytic vesicle-mediated protein trafficking (endocytosis, transcytosis, and/or recycling) [3–6]. The recycling and/or endosome-mediated degradation of integral membrane proteins at the apical ES further destabilizes spermatid adhesion, facilitating the release of sperm at spermiation. We believed these candidates and their signaling pathways will also provide insight into the role of retinoid signaling during spermiation and will suggest new targets for contraceptive validation.

Our current study also revealed that the aberrant F-actin organization is associated with diminished nectin-3 expression in both *Rara−/−* and pan-RAR antagonist-treated testes. As such, the downregulation of nectin-3 may affect the proper formation and/or maintenance of the actin scaffold at apical ES. Similar to what we have observed in *Rara−/−* and pan-RAR antagonist-treated testes, the apical ES of step 9 and 10 nectin-2-deficient elongating spermatids often lacked F-actin bundles. Afadin, which co-localizes with F-actin at apical ES, was also found to be absent in apical ES junction of nectin-2 null testes suggesting that nectin-2 recruits and/or maintains F-actin bundles at apical ESs through afadin [35]. Nectin-3, on the other hand, is essential for the localization of nectin-2 at apical ES, as suggested by the observation that nectin-2 was absent and the F-actin was disorganized in nectin-3 null testes [36]. In support of the critical roles of the cytoskeletal elements in proper F-actin bundles at the apical ES during normal spermiogenesis, it should be recalled that, similar to nectin-2 and nectin-3, disorganization of the actin cytoskeleton was also observed in junctional adhesion molecule-C (JAM*-*C)–deficient male mice, which exhibited male-specific infertility as well [47].

It has also been reported that changes in the phosphorylation status of tubulin can result in a failure to undergo spermiation [3]. Using an in vitro tubule culture system, it has been reported that the release of spermatids from cultured stage VIII tubules decreased when serine/threonine kinase activity was inhibited, yet was increased with the addition of okadaic acid, a serine/threonine phosphatase inhibitor [48]. Consistent with this, antibodies against phosphorylated serine, threonine and tyrosine proteins immunostain the Sertoli cell–spermatid junction during spermiation [48, 49]. Immunoprecipitation studies on protein interactions before and after spermatid disengagement suggest that ERK phosphorylation and 14-3-3 signaling may play important roles in spermatid release [48]. Lastly, phosphorylation of various adhesion molecules has been shown to be involved with spermiation [3, 48–51]. One of the most common posttranslational modifications is phosphorylation and indeed, phosphorylation of specific sperm proteins is an important event necessary to achieve fertilization [52].

We demonstrated in this study an abnormal localization of both tyrosinated and detyrosinated tubulins surrounding the retained spermatids in the seminiferous epithelium upon interference with retinoid signaling (Figure 6). Microtubule dynamics, specifically involved alteration of the levels of tyrosinated tubulin, which is highly expressed during tubulin polymerization, and detyrosinated tubulin, which is found in stabilized microtubules and shown to be important for anchoring elongating spermatids into the deep crypts of Sertoli cells at stages IX−XII. It has been suggested that the localization of tyrosinated tubulin in a crescent overlying the convex aspect of the acrosomal region of step 9–12 elongating spermatid heads was to allow the establishment of microtubules (tubulin polymerization) [43]. Once step 14–15 condensing spermatids at stages II−VI were in the crypts of Sertoli cells, tyrosinated tubulin became condensed into a spoke-like pattern, indicating active tubulin polymerization and establishment of microtubules to support the apical ES between the spermatids and Sertoli cells [43]. This is critical because it takes part in the relative arrangement of actin and microtubule cytoskeleton for spermatid translocation and spermiation. During stages VII−VIII, the spoke-like pattern of tyrosinated tubulin was weakly and sparsely detected, reflecting the fact that the spermatids have already been translocated to the luminal surface and the microtubule tracts are no longer needed. In contrast, in the absence of retinoid signaling, spermatids were not translocated and remained in Sertoli cell crypts, concomitant with the persistence of microtubule tract polymerization (Figure 6). Our observation of the presence of tyrosinated tubulins surrounding the retained spermatids was consistent with the notion that spermatid translocation from the adluminal to the luminal compartment does not occur in *Rara−/−* testes and pan-RAR antagonist-treated testes. Thus, retinoid signaling-induced changes in tubulin dynamics may play a role in defects during spermatid translocation toward the lumen. A recent finding demonstrating that dynein 1 supports spermatid transport and spermiation during spermatogenesis in the rat testis, using RNAi to knockdown cytoplasmic dynein 1 heavy chain and an inhibitor ciliobrevin D [40], suggests that it would be interesting to determine whether dynein is also affected upon inhibition of retinoid signaling.

In conclusion, we note that it is of interest that retained spermatids with detyrosinated tubulin were observed in the pan-RAR antagonist-treated testes, but not in the retained spermatids in *Rara−/−* stage IX^*^ tubules. This observation may indicate that long-term effects of RARA deficiency are associated with an absence of detyrosinated tubulin surrounding retained spermatids as well as with a difference in tubulin defects between acute and chronic lack of retinoic acid signaling. Retinoid signaling-induced changes in tubulin dynamics thus appear to play a role during spermatid translocation toward the lumen and spermatid release and proteins involved in this process may constitute new targets for male contraception.

## Supplementary data

Supplementary data is available at *BIOLRE* online.

## Supplementary Material

Chung_et_al_SupplFig_S1-S5--ALL-Final_ioaa123

## Notes

This work was supported by National Institutes of Health Grant U01-HD060479 and P50-HD093540 project 1 (to D.J.W. and S.S.W.C.).

## Notes

**Grant Support:** This work was supported by National Institute of Child Health and Human Development Grants U01-HD060479 and U54-HD093540/P50-HD093540, project 1 (to D.J.W. and S.S.W.C.).

## Notes

Disclosure Statement: The authors have nothing to disclose.
