# Supplementary material for: Filamentous actin disorganization and absence of apical ectoplasmic specialization disassembly during spermiation upon interference with retinoid signaling†
Source: Biol Reprod. 2020 Jul 17;103(2):378–89. doi: 10.1093/biolre/ioaa123 (PMC7401411; doi:10.1093/biolre/ioaa123)

Filamentous actin disorganization and absence of apical ectoplasmic specialization disassembly during spermiation upon interference with retinoid signaling

Sanny S. W. Chung[1], Nika Vizcarra[3] and Debra J. Wolgemuth[1-4]

Legends to Supplementary Figures

Supplementary Figure S1. Stage-specific localization of F-actin (green) at apical ectoplasmic specialization (ES) between developing spermatids and Sertoli cells in the seminiferous tubules. **A–H:** Histological sections of testes from 12-week-old adult wild-type mice. The F-actin bundles of the ES are labeled with fluorescent dye-conjugated phalloidin (green). **A-H**: Original magnification, x40. Roman numerals indicate the stage of the tubules.

Supplementary Figure S2. Absence of F-actin (green) at the apical ES of step 9 spermatids and aberrant location of F-actin surrounding the retained late elongated spermatids at stage IX tubules of *Rara-/-* testes. A–F: Histological sections of testes from 8-week-old adult control (**A, B**) and *Rara-/-* mice (**C-F**). The F-actin bundles of the ES are labeled with fluorescent dye-conjugated phalloidin (green) and counterstained with DAPI (blue). **A, C, E**: Original magnification, x60. Left panel (**B, D** and **F**) are the insert of right panel (**A**, **C** and **E**). **C** (merged image of F-actin and DAPI) and **E** (DAPI) showed the same tubule. Arabic numerals indicate the step of spermatid differentiation. Roman numerals indicate the stage of the tubules. Abnormal tubules are labeled with a Roman numeral followed by an asterisk (e.g. stage IX*). Yellow arrows in **C, E-F** point to the abnormally retained elongated spermatids while the red arrow in **D** indicates step 9 spermatids.

Supplementary Figure S3. The F-actin disorganization is associated with partially diminished nectin-3 expression in *Rara-/-* testes. A–D: Histological sections of testes from 12-week-old adult control (**A-B**) and 8-week-old adult *Rara-/-* mice (**C-D**). **A-D**: Original magnification, x60. **Left panel**: co-localization of nectin-3 (red) and DNA (blue); **middle panel**: co-localization of nectin-3 (red) and F-actin (green); **right panel**: merged figure of nectin-3 (red),

F-actin (green) and DNA (blue). Arabic numerals indicate the step of spermatid differentiation. The approximately staged abnormal tubules are designated by a Roman numeral followed by an asterisk (e.g. stage IX*).

Supplementary Figure S4. **Localization of tyrosinated tubulin and detyrosinated tubulin by confocal microscopy: A–B**, Localization of tyrosinated tubulin and detyrosinated tubulin in control stage IX (**A**) and V (**B**) tubules, counterstained with DAPI (blue) and PNA (white).

Supplementary Figure S5. **Localization of tyrosinated tubulin and detyrosinated tubulin by confocal microscopy: A–B**, Localization of tyrosinated tubulin and detyrosinated tubulin in control stage VIII (**A**) and pan-antagonist-treated stage VIII* (**B**) tubules, counterstained with DAPI (blue) and PNA (white). Roman numerals indicate the stage of the tubules. Abnormal tubules are labeled with a Roman numeral followed by an asterisk (e.g. stage IX*).

Supplementary Figure S1

WT (F-actin)

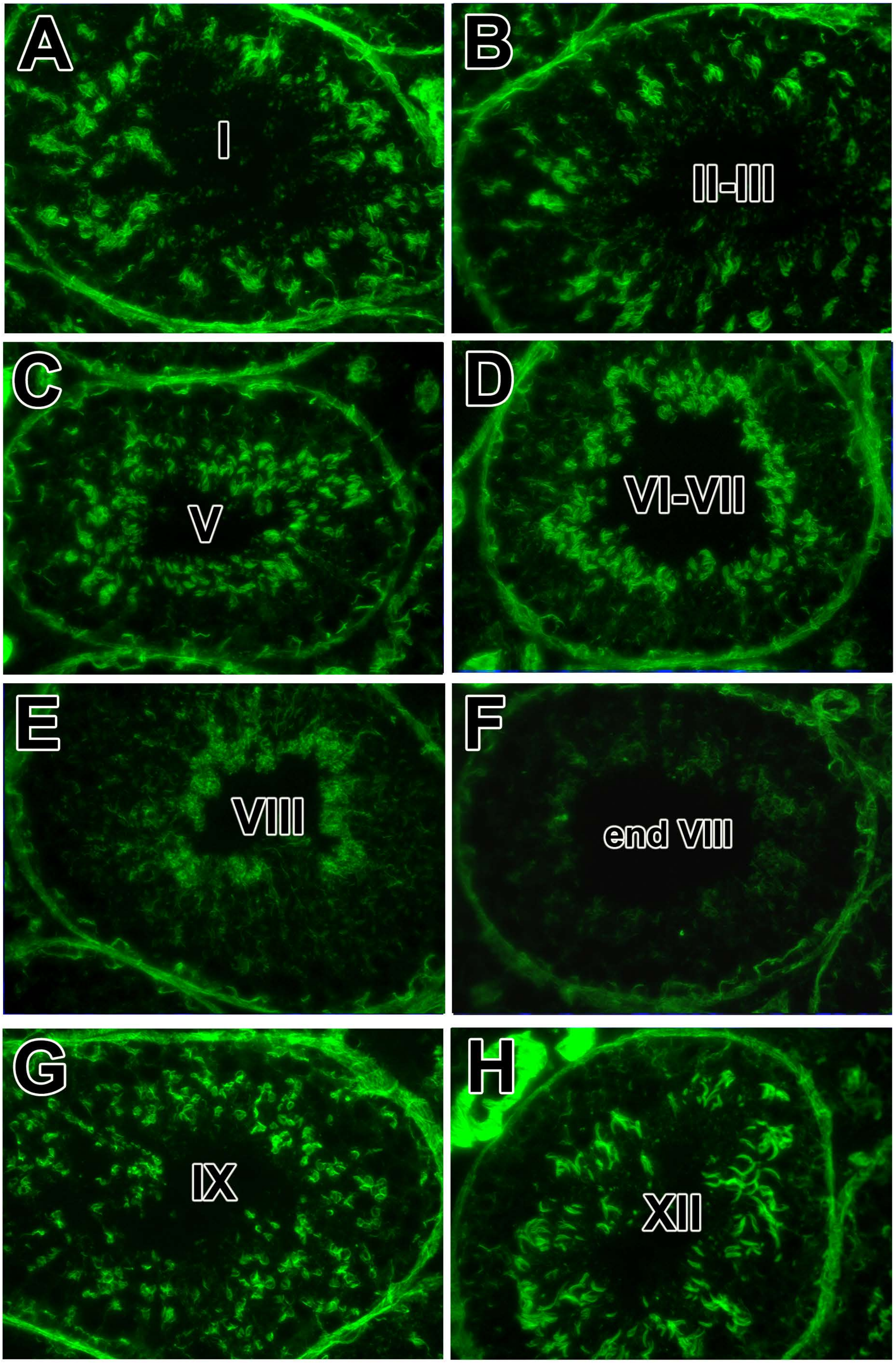

Supplementary Figure S2

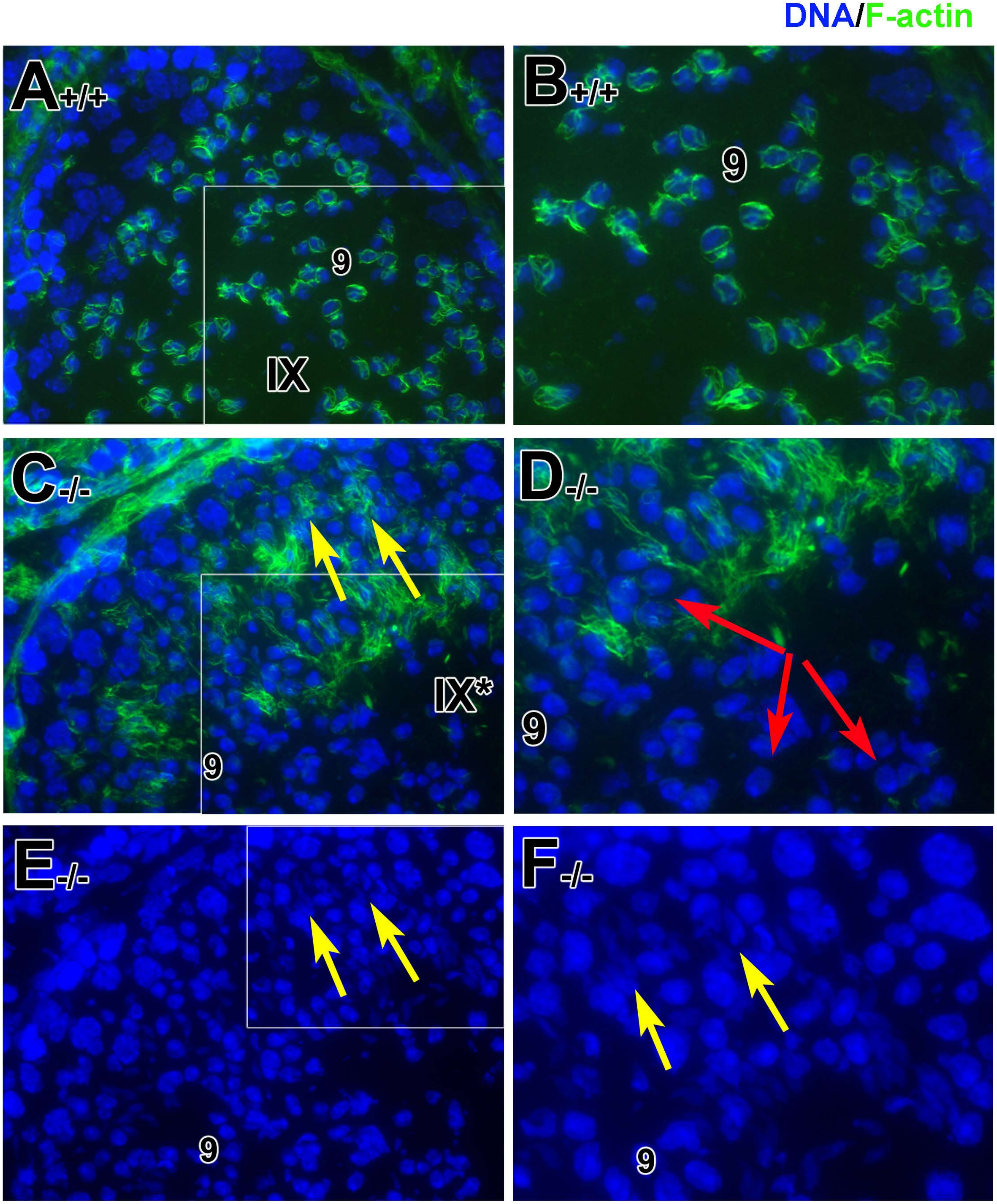

Supplementary Figure S3

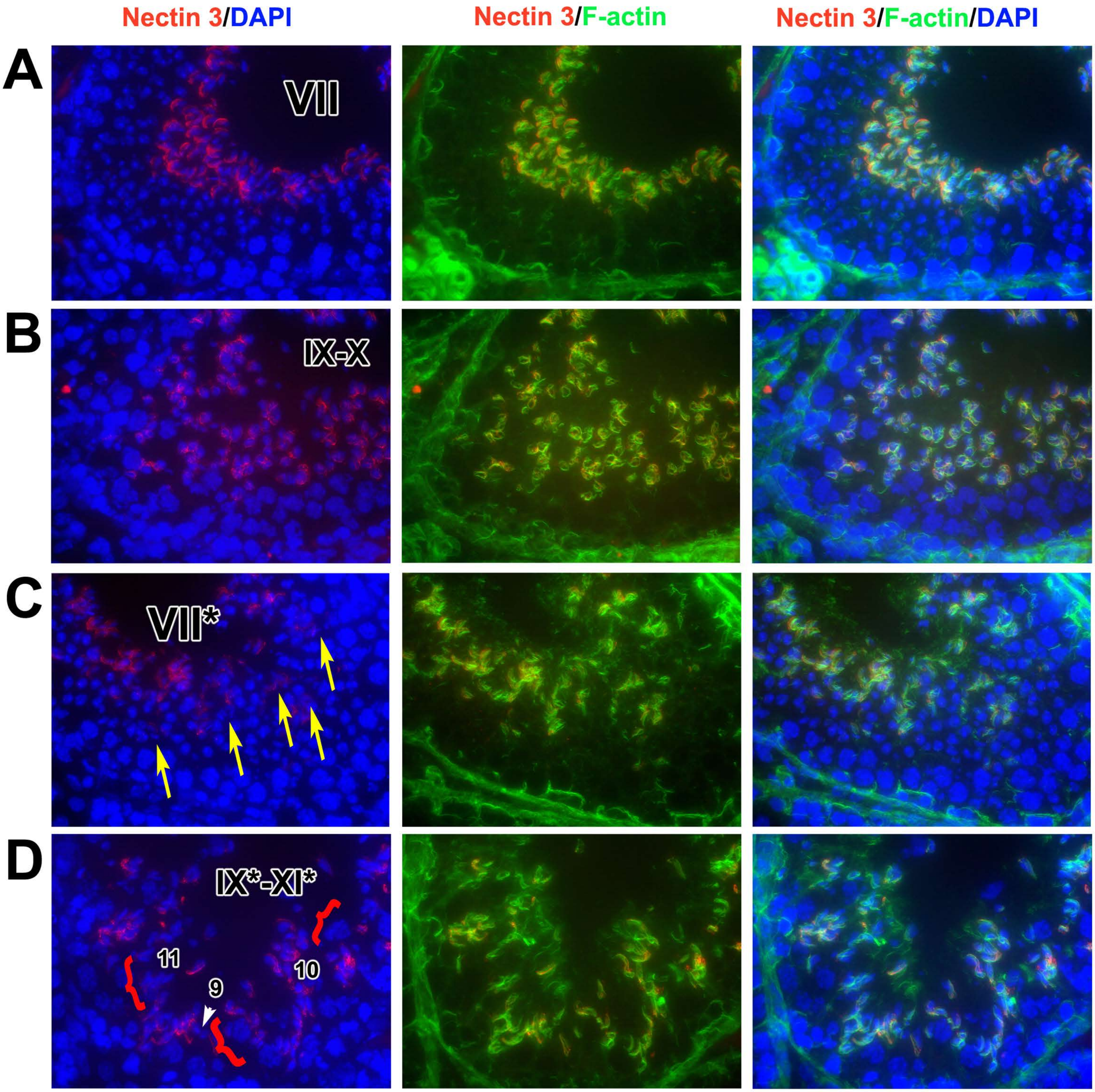

Supplementary Figure S4

A

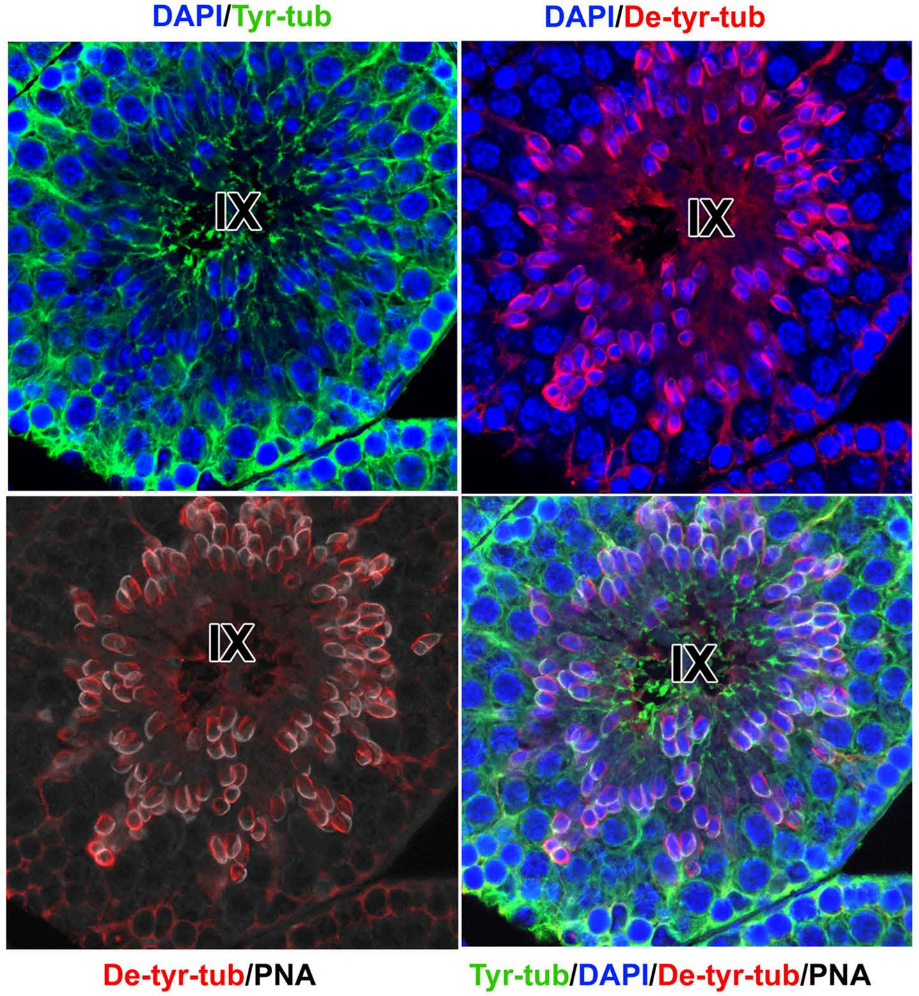

B

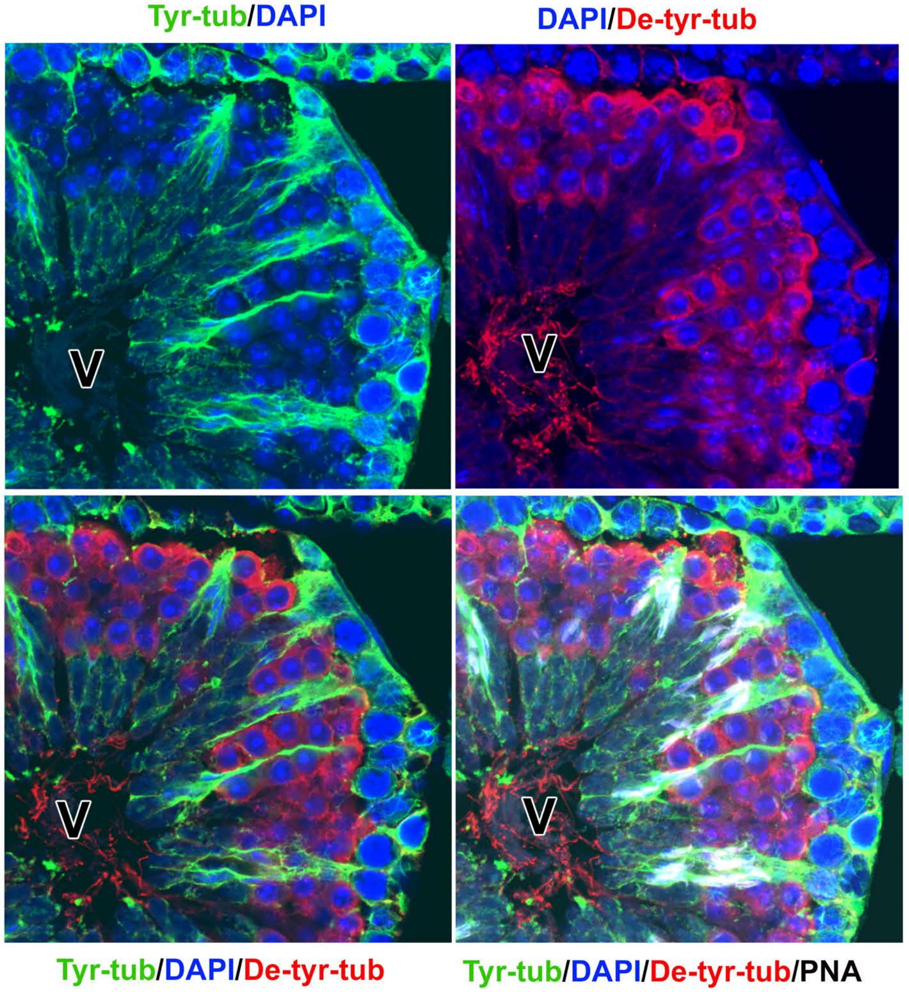

Supplementary Figure S5

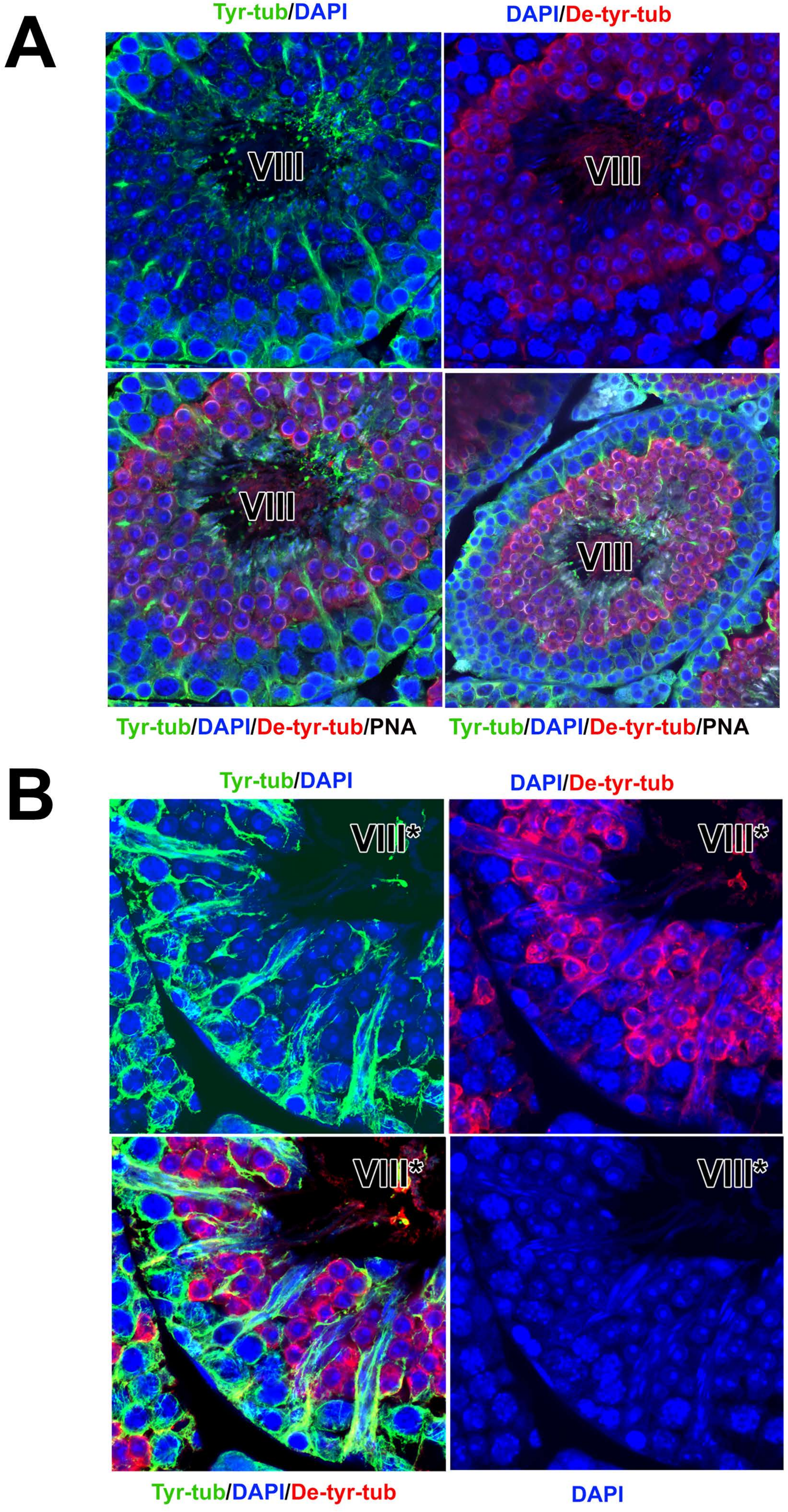

Supplement: Chung_et_al_SupplFig_S1-S5--ALL-Final_ioaa123 [file chung_et_al_supplfig_s1-s5--all-final_ioaa123.zip › Chung_et_al_SupplFig_S1-S5--ALL-Final_ioaa123.pdf]
